# Common cuckoo females may escape male sexual harassment by color polymorphism

**DOI:** 10.1038/s41598-019-44024-6

**Published:** 2019-05-17

**Authors:** Jin-Won Lee, Hae-Ni Kim, Sohyeon Yoo, Jeong-Chil Yoo

**Affiliations:** Department of Biology & Korea Institute of Ornithology, Kyung Hee University, 02447 Seoul, Republic of Korea

**Keywords:** Behavioural ecology, Sexual selection, Animal behaviour

## Abstract

Sexual conflict over mating rate is widely regarded as a selective force on the evolution of female-limited color polymorphism in invertebrates, such as damselflies and butterflies. However, evidence confirming its use in higher vertebrates remains limited. The common cuckoo, *Cuculus canorus*, is an avian brood parasite that does not provide parental care and represents a rare example of female-limited polymorphism in higher vertebrates. Specifically, males exhibit a monomorphic gray morph, while females are either gray or rufous colored, like juveniles. To test a prediction from the hypothesis that the rufous plumage of female cuckoos may help avoid excessive sexual harassment by males (the harassment avoidance hypothesis), we investigate color morph preference in male cuckoos. Mate choice experiments using playbacks of female calls with decoys mimicking both color morphs indicated that the attracted males immediately copulated with decoys without courtship displays, recognizing both color morphs as a sexual partner. However, the males attempted to copulate more frequently and excessively with the gray morph, which is consistent with the prediction from the harassment avoidance hypothesis. We propose that the absence of parental care augments sexual conflict over mating in cuckoos, resulting in the unusual evolution of female-limited polymorphism in this higher vertebrate.

## Introduction

Female-limited color polymorphism is considered to be an evolutionary strategy adopted by females to avoid excessive sexual harassment by males^1–5^. Sexual conflict over mating rates occurs widely during the process of reproduction^6,7^. Without doubt, selection favors multiple mating attempts by males. In comparison, the optimal number of mating attempts should be much lower for females, because their fitness is generally determined by the number of eggs they produce rather than the frequency of mating. This fundamental difference may result in females experiencing sexual harassment, including forced copulations by persistent males, which could have a detrimental effect on the survival and lifetime fitness of females^7–13^. Examples of costs imposed on females include the loss of time and energy for foraging^13–19^, increased risk of injury and sexually transmitted diseases^20–23^, and increased exposure to predation^24,25^. Furthermore, the consequences of sexual conflict might be more serious in promiscuous species that do not invest in parental care since the prolonged mating period may increase the duration of exposure of females to extreme sexual harassment^10,26^. This may explain why female-limited polymorphism is widespread in invertebrates with those breeding systems, such as damselflies and butterflies^1^.

In higher vertebrates, such as birds and mammals, female-limited polymorphism occurs relatively rarely. For example, in birds, only 23 species have female-limited polymorphism, representing approximately 0.2% of all bird species^27^. The common cuckoo (*Cuculus canorus*) belongs to one such species in higher vertebrates^28–30^. Adult males are monomorphic gray in color, whereas adult females have two color morphs, either gray or rufous (hepatic) with various intermediate forms^29^. Of note, rufous females are similar to juvenile cuckoos in general appearance, although they are never identical in every details^29^. In fact, rufous morphs occur more frequently in the juveniles of both sexes compared to adult females^29,31,32^.

The common cuckoo is an avian brood parasite that lays eggs into the nests of other species (hosts) and leaves the hosts to provide full parental care for their progeny at the expense of the host’s own chicks^33,34^. This phenomenon generates strong selection pressure on both interacting species to maximize their own fitness, making it a fantastic model system for the study of coevolution between species for decades^28,33,35–37^. Thus, the occurrence of sex-limited polymorphism in cuckoos has been naturally explained from the perspective of coevolution. For example, it is well established as an example of Batesian mimicry that the cuckoos resemble *Accipiter* hawks as a model, thereby increasing the protective benefits while parasitizing host nests^38–40^. However, the defence of mimicry could become less successful due to either improved awareness of host population or the rarity of models, which may lead to the evolution of polymorphism in cuckoos as a result of mimicry of different birds of prey (e.g., kestrel mimicry by the rufous morph)^29,32^. Thorogood and Davies^38^ showed a significant correlation between the occurrence of female polymorphism and hawk features among parasitic species belonging to Cuculinae. However, empirical data supporting the kestrel mimicry hypothesis are rare^32,39^. Alternatively, but not mutually exclusively, female cuckoos take advantage of breaking a parasite image formed in host populations by plumage color polymorphism^41,42^. For example, in a cuckoo population where gray females outnumber rufous females, host populations might be more alerted to the gray morph by the formation of parasite image (like “search image” in predator-prey systems^43^) acquired through social information and individual experience^41,42^. Therefore, hosts might be less likely to recognize the rufous female, a rarer morph, as a brood parasite, increasing the chance of successful parasitism over gray morphs through frequency-dependent benefits^41,42^. As such, most work on female-limited plumage polymorphism in cuckoos has focused on hosts being the main selective agent, and invoked hypotheses from predatory-prey coevolution. However, the possibility that these color polymorphisms arise because of sexual conflict has yet to be considered in cuckoos, although it is widely invoked as a selective agent for the evolution of female-limited color polymorphism in some other organisms.

Here, we propose an alternative hypothesis that the rufous females evolve to avoid persistent male sexual harassment in the common cuckoo (the harassment avoidance hypothesis) through the mimicry of juvenile morphs. Due to the parasitic habit of the common cuckoo, it could be characterized by a promiscuous mating system with a prolonged mating period and no parental care^44–46^. Thus, this species represents a rare example of this phenomenon in higher vertebrates, as nearly all species of higher vertebrates provide some form of parental care. These circumstances might augment sexual conflict over optimal mating rates (mate-searching males versus sire-seeking females), leading to excessive copulatory attempts by males^6,10,26^. Potential costs to female cuckoos might be substantial, as they includes those already stated plus the loss of time and energy searching and observing host nests, which is essential for their fitness. In fact, flying female cuckoos being chased violently by two or more persistent males is commonly observed, and often end in copulation and sometimes multiple males and females scrambling for mating^45^ (JWL, personal observation). Therefore, it could be assumed that all females would be chased by males while siring their eggs, but not all females would be detected by hosts while parasitizing their nests since hosts often leave their nests during the laying period. It indicates that if color is subject to selection, the selection pressure from male harassment and excessive mating rates might be stronger than that from the interaction with hosts.

Using playbacks of female calls with decoys mimicking both color morphs, we experimentally tested a prediction from the harassment avoidance hypothesis that rufous color may help reduce persistent male sexual harassment. The adaptive mate choice theory predicts that when one sex is polymorphic, morph preference by the other sex should differ according to the relative fitness values of each morph^47,48^. In this context, previous hypotheses based on the coevolutionary perspective predict that males should prefer the rarer morph in a population (the rufous morph in our population), because the rarer morph has more chance of parasitism success and, thus, more fitness benefits^41,42,49^. In contrast, the harassment avoidance hypothesis predicts that compared to gray morphs, rufous females should receive less sexual attention from males because of their juvenile appearance and/or associated costs^32,50^.

## Results

### Cuckoo response and control experiments

**Figure 1:**
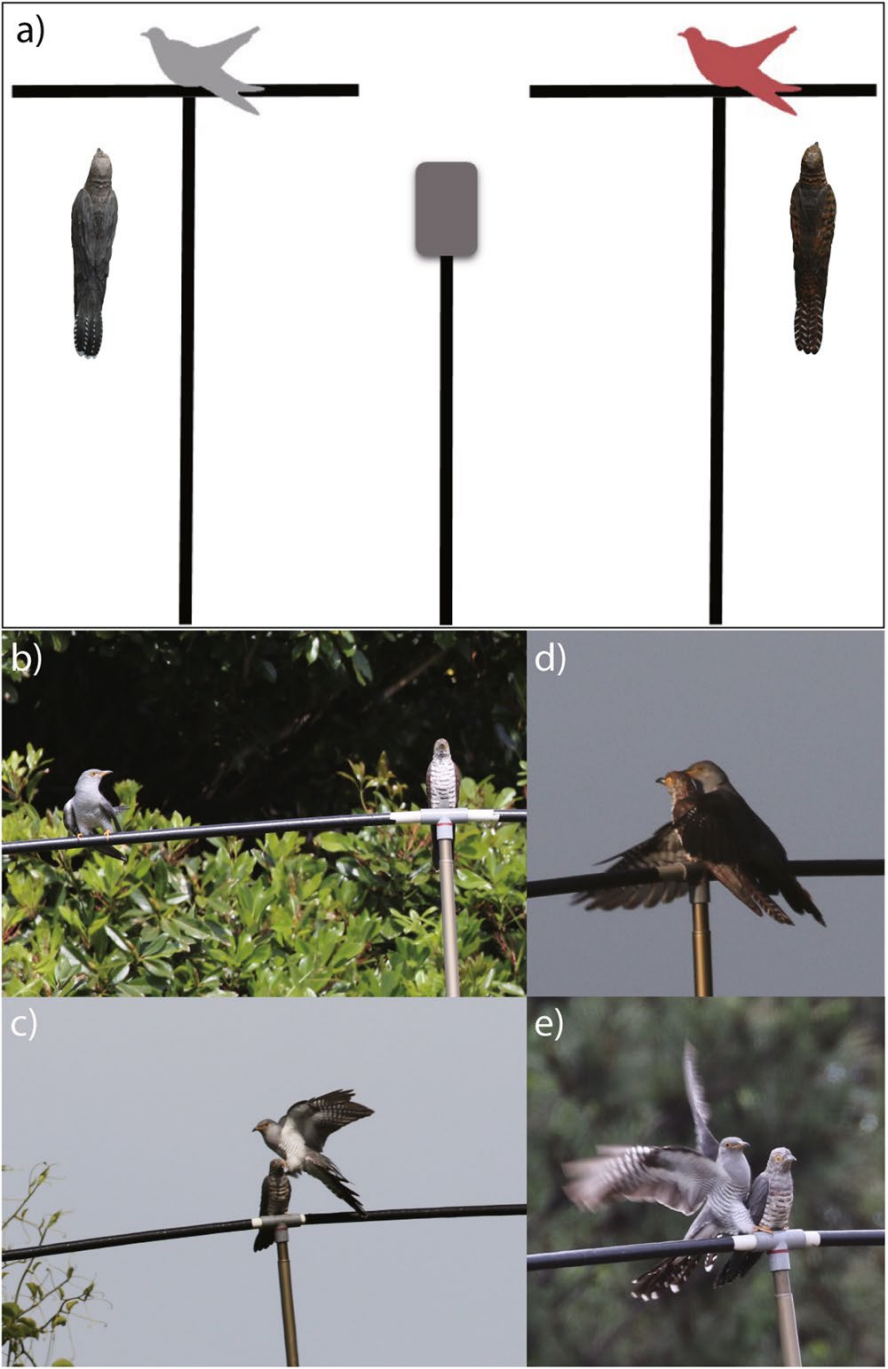
Experimental set-up and cuckoo responses. (**a**) Experimental set-up and images of decoys used in the experiment. The gray quadrangle in the middle represents a speaker with an embedded mp3 player. See the text for details. (**b**~**e**) Types of cuckoo responses. In response to the experiment, cuckoos either perched on the bar (**b**), touched a decoy (**c**), or attempted to copulate with decoys: with a rufous morph (**d**) and with a gray morph (**e**).

Once the cuckoos responded to the playback of female calls, they showed one of three response behaviors: perching on the horizontal pole, attempting copulation with a decoy, and touching a decoy (Fig. 1, Supplementary Video). We assumed that the cuckoos selected a female morph if they showed any of these behaviors to a decoy. We identified which female morph was selected first and how often each morph was selected for the 20 min after the first selection response. We also analyzed copulation attempts separately from the overall selection response, as this activity is the most biologically meaningful in terms of mating behavior.

**Figure 2:**
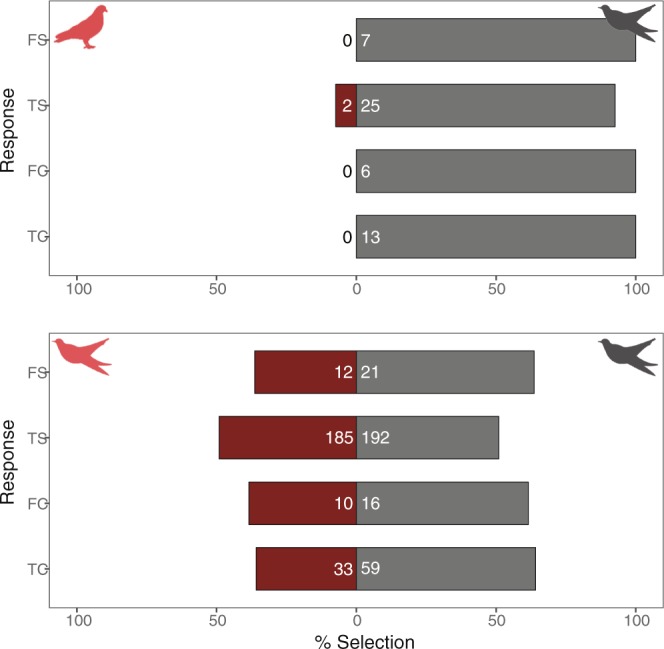
Response of male cuckoos to the control (upper) and main (lower) experiments. FS, TS, FC, and TC represent first selection, total number of selections for 20 min after the first response, first copulation, and total number of copulation attempts, respectively. Different symbols represent decoys mimicking different species (dove or cuckoo), while different colors represent different color morphs (gray or rufous). The numbers of FS and FC represent the number of individuals, while the numbers of TS and FC indicate the sum of number of selection/copulation attempts made by all individuals.

Control experiments that used decoys mimicking the gray female cuckoo and the rufous-turtle dove, *Streptopelia orientalis*, a common species in our study area, demonstrated that cuckoos reacted selectively between the morphs in response to the playback of female cuckoo calls (Fig. 2a). In seven control experiments, male cuckoos consistently selected first and, almost exclusively, the dummy cuckoo (first selection: binomial test, p = 0.016; total selection: Wilcoxon signed-rank test, V = 28, p = 0.022), and all copulation attempts occurred with the dummy cuckoo only (first copulation: binomial test, p = 0.031; total copulation: Wilcoxon signed-rank test, V = 21, p = 0.034). These results confirmed that our experimental design and process could effectively determine the preference of cuckoos for/against the gray and rufous female morphs.

### Male responses to experiments

**Figure 3:**
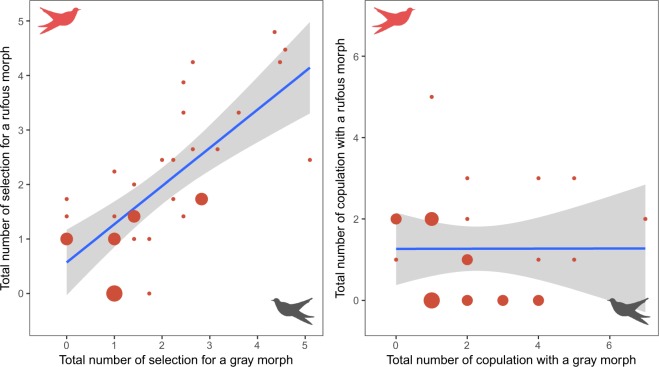
Scatter plots with fitted lines and 95% confidence intervals, showing the correlation between the gray and rufous morphs for the total number (square-rooted) of selections (left) and the total number of copulations (right). Circle size varies according to sample size.

Male common cuckoos (n = 33) tended to select the gray morph preferentially over the rufous morph with different statistical significance levels (Fig. 2b). The first selection and copulation attempt appeared to occur preferentially at the gray morph, albeit not significant (binomial test: first selection, p = 0.16; first copulation, p = 0.32). During 20 min experimental time, 33 males made 377 selections in total without preference to specific morph (185 for gray vs. 192 for rufous; Wilcoxon signed-rank test: V = 210, p = 0.88). Significant difference was found in the total number of male copulation attempts (Wilcoxon signed-rank test, V = 240, p = 0.036), with the gray morph receiving almost double the number of copulation attempts as the rufous morph (Fig. 2). This difference was clear when we compared the pairwise relationship between the two morphs for the total number of selections and total number of copulations (Fig. 3). There was a strong positive correlation between the gray and rufous morphs for the total number of selections received from male cuckoos (Spearman’s rank correlation: *r*_s_ = 0.74, p < 0.0001, Fig. 3a), indicating that males shuttled between the two morphs during experiments. For the total number of copulations, however, this correlation disappeared (*r*_s_ = 0.26, p = 0.14), with the gray morph receiving more copulation attempts from male cuckoos, implying that the gray females receive more sexual harassment from males than did rufous females. Male morph preference was not associated with temporal factors, such as time of day, date, and year (Supplementary Table 1).

### Female responses to experiments

We found that female cuckoos were aggressive to other individuals, like males. Although our experiments did not target females, four female cuckoos were attracted to and responded to the playback (Supplementary Table 2). Three females selected the gray morph first, with all three individuals making one to three visits only and watching the decoy on the bar without making physical contact. The female that selected for the rufous morph first made nine approaches with some aggressive physical contact (Supplementary Table 2). However, copulation attempts, as expected, was never made by any of the females.

## Discussion

Our study demonstrated that male cuckoos recognize both the rufous and the gray morphs as potential sexual partners, which may explain how the rufous females exist in the breeding population. However, we found that males tended to choose the gray morphs first and attempted to copulate with them more persistently than they did with the rufous morphs. Of note, male cuckoos attempted to copulate with the decoy in response to the playback of female calls, without any courtship display or consent of females. Thus, gray females appear to suffer more severely from excessive mating attempts by persistent males than did rufous females. Rufous female cuckoos are extremely rare in our study populations^50^; thus, these male responses are clearly not consistent with the prediction from the previous hypotheses based on the coevolutionary perspective, which predicts that males should prefer a rarer morph, the rufous one in our study. Instead, observed male preference for female color morph was consistent with that expected from the harassment avoidance hypothesis; that is, rufous plumages may help reduce extreme male harassments.

The evolution of female polymorphism to counteract male sexual harassment could be explained via two different routes. First, as we already proposed, the alternative form could be evolved by mimicking juveniles (juvenile mimicry hypothesis), which is corresponding to male mimicry hypothesis proposed in some butterflies and damselflies^1,51,52^. Juvenile mimicry could be achieved either genetically as a fixed form (e.g. genetic polymorphism^53^) or through developmental phenotypic plasticity (e.g. paedomorphism^54^), with no clarification in the common cuckoo yet. However, the resemblance of females to sexually immature juveniles might reduce the likelihood of males chasing and copulating persistently with them, irrespective of the mechanism. Alternatively, compared to gray females, rufous females might have lower fitness values, making them less attractive to males. The lower fitness values are if the rufous females become subordinate to the gray females as a result of juvenile mimicry in a dominance hierarchy that is closely associated with the availability of host nests (^44,45^, but see^55^). A previous study showed that female cuckoos retain high levels of testosterone during the breeding season^56^, implying the presence of antagonistic interactions among females. Our experiments also showed that some female cuckoos respond aggressively to the presence of other females. It would be interesting to see how much female-female competition can explain the evolution of plumage polymorphism in cuckoos^50^.

Alternatively, females could escape male attention simply by evolving a non-mimetic, different phenotype from the common morph. The learned mate recognition hypothesis^57–60^ explains that males prefer to mate with females of a more common morph because frequent exposures to the common morph allows males to recognize it as a potential mate more easily through an analogous process to predators forming a search image for the most common prey morph^61^. Accordingly, females of a common morph than a rare morph are more likely to be sexually harassed by males, incurring larger fitness costs. Such frequency-dependent fitness trade-offs between alternative morphs might maintain their relative frequency at some equilibrium point, given no other selection pressures. In our study populations, where rufous females are extremely rare^50^, the learned mate recognition hypothesis predicts that male cuckoos should prefer to mate with the gray females. Key differences from the juvenile mimicry hypothesis are that this hypothesis considers the rufous morph to be a simply different form from the gray morph, with no frequency-independent costs/benefits according to color (e.g. costs that arise by mimicking juveniles). Furthermore, male mate preference would not be innate, but changeable, according to female morph frequency^57,58^.

Although our results were generally consistent with the learned mate recognition hypothesis, several lines of evidence hinder us from adopting it. First, this hypothesis could generate predictions with respect to the probability of recognizing and locating a specific morph as a mate, but does not explain how persistently males should mate with females of a specific morph. In our study, both gray and rufous females were recognized as mating partners by males that exhibited no significant difference in the first selection. Instead, a significant difference was found in how persistently males attempted to copulate with a specific morph (i.e., total number of copulation attempts). Second, it is difficult to explain based on this hypothesis why the rufous females are mostly minor in number in most populations and have rarely been observed in our study site^31,50^. Under the learned mate recognition hypothesis, the frequency of the two morphs should fluctuate at some equilibrium points, without wiping out any one morph, even though the frequencies might be biased to one morph, to some degree, by the presence of other mechanisms, such as color-dependent predation rates^62^.

The juvenile mimicry hypothesis better explains the rarity of the rufous morph in our study populations, along with the variable morph frequencies observed across other populations of the common cuckoo and other *Cuculus* species^31,50^. The juvenile mimicry hypothesis assumes the presence of frequency-independent costs imposed on the rufous morph, which probably arose because of juvenile mimicry. As a result, in the absence of extreme sexual harassment by males, the gray morph is always more adaptive than the rufous morph. However, as male harassment is excessive, these costs might be offset if the benefits they accrue from escaping male harassment are large enough. Alternatively, we cannot rule out the possibility that both hypotheses may explain together the occurrence of female polymorphism in cuckoos. Specifically, for example, juvenile mimicry may work effectively when the rufous females are minor; however, as their numbers increase, males are likely to learn and recognize them as a sexual partner, and thus their mimicry become less effective with regard to avoiding male harassment. Comparing color morph preferences in male cuckoos obtained from multiple populations with different female morph ratios may disentangle these alternative hypotheses.

Both hypotheses assume that morph frequency could be regulated by the strength of selection exerted by male harassment. Several biotic and abiotic factors might influence the strength of male harassment, both spatially and temporally. First, for example, as the number of interacting individuals increases, the strength of male harassment that females experience might increase (density). Second, the sex ratio of populations might affect sexual harassment. For instance, females might suffer more severely in male-biased populations (sex ratio). Third, females in open habitats, like reedbeds, might be chased by males more easily and persistently than those in closed habitats, like woodland (habitat characteristics). Finally, the strength of male harassment might be influenced by the social system that regulates the spacing behavior of individuals. For example, exclusive male territories might cause the level of harassment that females receive to decline (social system). Collectively, these factors might determine the intensity of selection pressure on females to evolve polymorphism and generate variable morph frequencies within and among *Cuculus* species, which needs to be explored in future studies.

A recent study of vocal activity in a population of parasitic cuckoos showed that female vocal activity is highly structured across seasons, rapidly peaking within approximately one month of arrival, after which it decreases (Yoo *et al*. under revision). Since female calls elicit male copulation attempts, sexual activity, such as male chase and mating, might occur intensively during the early breeding season. Mate choice mechanisms are poorly understood in cuckoos; however, females might generate calls and elicit chasing by males intentionally to access male qualities for mating during this period. Secretive female behavior during the rest of the breeding season might occur not only to enhance parasitism success, as explained from the coevolutionary perspective^33^, but also to avoid unwanted copulation attempts and sexual harassment by males. This hypothesis could be strengthened by the fact that female birds are able to store live sperms in their genital track for certain periods of time^63^. If so, it seems adaptive for female cuckoos to acquire sperms during the early breeding season and use it for subsequent clutches through the proximate breeding season, as this would save time for searching and observing host nests by avoiding repeated mating with males. That is, females might avoid male harassment in several ways: namely, morphologically by mimicking juveniles, behaviorally by being secretive, and possibly by storing sperms.

In conclusion, the results of our study suggest that strong sexual conflict over mating rates, which is closely associated with the absence of parental care, might have an analogous effect from invertebrates to higher vertebrates on the evolution of morphology, behavior, and social systems. Considerably more works will need to be done to comprehensively verify the harassment avoidance hypothesis in the evolution of female-limited polymorphism in brood parasitic cuckoos. First, it is fundamentally necessary to determine how much male harassment actually reduces female fitness in cuckoos, although it is likely to be extremely difficult to measure in the field. Second, although coloration is similar between rufous females and juveniles, it does not necessarily mean that the appearance of rufous females is identical to rufous juveniles. In fact, there is a number of distinctive features of juveniles (e.g. white patch on the nape, and less distinct wing barring). In addition, there is a large variation in coloration among rufous females in *Cuculus* species^29,50^. Therefore, experimental studies to test male responses toward juveniles as well as females with various degree of rufous coloration are needed to verify juvenile mimicry as a means of harassment avoidance. Third, it needs to test whether or not the harassment avoidance hypothesis can explain the significant correlation between the occurrence of female polymorphism and hawk mimicry across parasitic species in Cuculinae^38^. Nevertheless, we advocate that avian brood parasitism could represent an ideal model system to test how the absence of parental care is associated with the evolution of phenotypic diversity, sexual relationship, and social structures in higher vertebrates, which could broaden our understanding of the universal effects of non-parental care.

## Methods

### Study Site

Field experiments were conducted from May to June in 2015 to 2017 in the eastern area of Jeju-do, which is the largest island (ca. 1,848 km^2^) in the Republic of Korea (33° 31′N, 126° 32′E). The common cuckoos at Jeju-do mainly parasitize the meadow bunting, *Emberiza cioides*, and so remain in the area from early May to early August^64^. However, most breeding activity appears to occur in May and June. Previous studies suggested that the rufous female morph is extremely rare in our study population^50^. Furthermore, although some female cuckoos have a rufous tinge on their neck and shoulder, most females are completely gray, like the males^50^.

### Male mate choice experiments

We investigated male color preference for the two female color morphs. We used two 3D-printed decoys that virtually resemble female common cuckoos in size and appearance, but were different in color (gray versus rufous), mimicking the gray and rufous female morphs (Fig. 1a). Plastic models instead of taxidermic cuckoo mounts were used because, using 3D printing technology, it was possible to use the identical appearance of decoys, ensuring that variation in responses stemmed from differences in colors rather than in shape. Additionally, plastic decoys are more portable and durable against harsh field conditions (e.g., high humidity, rain, strong sunlight, wind) and physical contacts with experimental birds. However, the static models could never replicate live specimens, as they do not behave and react, and so male responses might be overestimated. Furthermore, it should be noted that there is no experimental study testing whether or not cuckoos respond similarly to artificial color of models as they respond to real feather colors of live cuckoos, and colors visible to human eye might not be perceived in the same way as birds, since they have superior light perception, extending the range of color vision into ultraviolet (UV)^65^. Nevertheless, previous studies have shown that static taxidermic or wooden cuckoos with artificial coloration could elicit similar responses from experimental birds to live cuckoos (e.g.^41,42,66^), while the gray and rufous feathers of common cuckoos do not appear to reflect UV strongly^31^.

For the experiment, we searched and located free-ranging male cuckoos over the wide area (approximately 250 km^2^) based on their calls and after the experiments finished, we moved to another distant place. Our cuckoo-capture data of multiple years (2013–2018) show that recapture rates within a breeding season as well as between breeding seasons were extremely low with this method (both 0%, JWL unpublished data), indicating that it is unlikely that the same individuals were tested repeatedly. Once an experimental male was chosen, we simultaneously presented it with the gray and rufous cuckoo decoys, while attracting it by using female playback calls. We set up two T-bars in the male’s range, which were separated by 5 m. Each T bar was made of a 5 m high vertical pole and 2 m long horizontal pole across it (Fig. 1a). We also erected another pole of 5 m height or less with a speaker that had an embedded mp3 player in the middle between the two T-bars (Fig. 1a). We then randomly allocated the gray or rufous cuckoo decoys to each T-bars, fixing them to the middle of horizontal poles, facing the same direction and with the same posture. Finally, we used the playback of female calls to attract experimental cuckoos. Female calls were played randomly and repeatedly at a constant volume. Several different calls were used that were recorded from different females in Jeju-do. Female cuckoos produce calls infrequently in nature and, thus, our playback might overestimate male responses. Nevertheless, this method could be an efficient approach for inferring male preference between the two color morphs. The attracted cuckoos usually first fly around the experimental equipment (i.e., two T-bars, a speaker pole), perch near the equipment, and then approach the decoys. Therefore, the experimental equipment was placed near trees or something on which birds could perch to ensure that the male cuckoos watch both decoys before they choose a female. Observations were made from the furthest possible distance (usually >50 m) to avoid any disturbance by the observers and were also filmed using a video camera to double check. The response behavior of cuckoos was divided into three categories (i.e., perching on the horizontal pole, attempting copulation with a decoy, and touching a decoy), and was quantified for 20 min after the first response behavior occur.

To validate the results of our experiments, we also conducted control experiments that had the same process but presented the male with the gray cuckoo decoy and a decoy mimicking the rufous-turtle dove, *Streptopelia orientalis*, a common species in our study area. All fieldwork and experimental procedures were approved by Kyung Hee University Animal Ethics Committee, and were performed in accordance with relevant national and international guidelines and regulations (e.g. experimental time per individual).

### Data analysis

Cuckoos usually repeatedly fly in and out of T-bars in response to the playback of female calls; thus we quantified any response behavior (i.e., perching, touching and copulating) occurring between flying in and out, defining it as a bout of behavior. When individuals perform successive behavior in a bout (e.g., first perching on the pole and subsequently copulating or touching), we recorded the behavior that showed the largest intensity of sexual approach to a decoy; that is, copulating had priority over the others, while touching had priority over perching. Cuckoos choose a specific female color morph if they showed any of these response behaviors to a decoy, and quantified which female morph was selected first and how many times each morph was chosen repeatedly for 20 min after the first selection response. Copulation attempts, due to their significance in terms of mating behavior, were also analyzed separately from the overall selection response. However, we did not analyze touching behavior separately, as it may simply occur due to a lack of reaction from the static model.

We used the binomial test to assess for which female color morph the first selection or copulation attempt occurred. Differences and correlations between the gray and rufous morphs for the total number of selections and the total number of copulations over 20 min were analyzed by the Wilcoxon signed-rank test and Spearman rank’s correlation, respectively. We employed generalized linear models (GLMs) with binomial error structure to check any temporal effect (i.e. experiment start time, date, and year) on male response behavior (i.e., first selection, total selection, first copulation, total copulation). For each of the response variables, we first fit a maximal model with these three explanatory variables, and then we removed non-significant terms to obtain a minimal adequate model with significant terms only^67^. Model coefficients were presented with 95% confidence intervals (CI). All statistical tests were performed in R version 3.5.0^68^.

## Supplementary information

Supplementary video

Supplementary tables

## Data Availability

The datasets generated during and/or analysed during the current study are available from the corresponding author on reasonable request.
