# Supplementary material for: Common cuckoo females may escape male sexual harassment by color polymorphism
Source: Sci Rep. 2019 May 17;9:7515. doi: 10.1038/s41598-019-44024-6 (PMC6525237; doi:10.1038/s41598-019-44024-6)
Supplement: Supplementary file 2 — Supplementary tables [file 41598_2019_44024_MOESM2_ESM.docx]

Supplementary Information

**Common cuckoo females may escape male sexual harassment by color polymorphism**

Jin-Won Lee^1,*^, Hae-Ni Kim^1^, Sohyeon Yoo^1^, Jeong-Chil Yoo^1^

^1^ Department of Biology & Korea Institute of Ornithology, Kyung Hee University, Seoul 02447, Republic of Korea

Supplementary table 1. Multivariate GLM results testing the effect of time of day, date, and year on color morph preference in male cuckoos.

|  | First selection | | | Total selections | | | First copulation | | | Total copulations | | |
| --- | --- | --- | --- | --- | --- | --- | --- | --- | --- | --- | --- | --- |
|  | ES | 95% CI | *P* | ES | 95% CI | *P* | ES | 95% CI | *P* | ES | 95% CI | *P* |
| Time | -0.16 | -0.37~0.01 | 0.09 | 0.05 | -0.01~0.11 | 0.08 | -0.18 | -0.43~0.03 | 0.12 | 0.02 | -0.08~0.12 | 0.66 |
| Date | -0.04 | -0.13~0.06 | 0.46 | 0.01 | -0.02~0.04 | 0.55 | -0.06 | -0.19~0.03 | 0.25 | 0.01 | -0.06~0.07 | 0.84 |
| Year | -0.86 | -1.93~0.08 | 0.09 | -0.01 | -0.29~0.28 | 0.97 | -0.14 | -1.26~0.91 | 0.8 | -0.09 | -0.64~0.44 | 0.75 |

Supplementary table 2. Responses of females to the experiments

| Female ID | First Selection | Total Selection | | Total Touching | | Total Perching | |
| --- | --- | --- | --- | --- | --- | --- | --- |
|  |  | Gray | Rufous | Gray | Rufous | Gray | Rufous |
| F1 | Gray | 1 | 1 | 0 | 0 | 1 | 1 |
| F2 | Rufous | 5 | 4 | 4 | 1 | 1 | 3 |
| F3 | Gray | 1 | 0 | 0 | 0 | 1 | 0 |
| F4 | Gray | 3 | 0 | 0 | 0 | 3 | 0 |
